# The atherogenic index of plasma and triglyceride-glucose index as promising predictors of overall and disease-free survival in postoperative breast cancer patients

**DOI:** 10.3389/fendo.2025.1728451

**Published:** 2026-01-06

**Authors:** Zhimin Chen, Minhui Luo, Longdan Liu, Mingwen Cheng, Honglan Gao, Shuangshuang Ma

**Affiliations:** 1 Department of Clinical Nutrition, The Yancheng Clinical College of Xuzhou Medical University, The First People’s Hospital of Yancheng, Yancheng, China; 2 Department of Plan and Quality Management, Ganzhou People’s Hospital, Ganzhou, China; 3 Department of Dermatology, The Yancheng Clinical College of Xuzhou Medical University, The First People’s Hospital of Yancheng, Yancheng, China; 4 Department of Public Health, Yancheng Center for Disease Control and Prevention, Yancheng, China; 5 Department of Clinical Nutrition, The Yancheng School of Clinical Medicine of Nanjing Medical University, Yancheng Third People’s Hospital, Yancheng, China

**Keywords:** atherogenic index of plasma, breast cancer, disease-free survival, insulin resistance, overall survival, triglyceride-glucose index

## Abstract

**Background:**

Insulin resistance (IR) is closely linked to breast cancer development and prognosis. This study aimed to evaluate the prognostic value of four surrogate indices of IR, the atherogenic index of plasma (AIP), triglyceride-glucose (TyG) index, TyG index combined with body mass index (TyG-BMI), and metabolic score for IR (METS-IR), for overall survival (OS) and disease-free survival (DFS) in postoperative breast cancer patients.

**Methods:**

This retrospective cohort study included 298 patients with primary breast cancer who underwent radical surgery. We used multivariable Cox regression, Kaplan-Meier analysis, restricted cubic splines, receiver operating characteristic (ROC) curves, and subgroup analyses to assess associations and predictive performance for 3-, 5-, and 10-year survival. Random survival forest analysis ranked the importance of IR-related variables.

**Results:**

In fully adjusted models, elevated AIP and TyG index were significantly associated with worse OS, with hazard ratios of 2.28 and 2.82, respectively, for the highest versus lowest tertiles. Per 1-standard-deviation (SD) increase in AIP and TyG was associated with a 50% and 55% increased risk of OS, respectively. For DFS, only the TyG index showed a statistically significant association in the tertile comparison. Notably, Kaplan-Meier analysis and multivariable Cox models consistently indicated significant associations for AIP and TyG with OS and DFS. The predictive performance of AIP and TyG as assessed by ROC analysis was similar to each other but superior to TyG-BMI and METS-IR across all time points. Random survival forest (RSF) analysis identified AIP and TyG as the most important variables for predicting OS and DFS, respectively. Subgroup analyses revealed consistent trends for AIP and TyG across different patient subgroups.

**Conclusion:**

Our findings suggest that the AIP and TyG index are promising, easily obtainable biomarkers for risk stratification and long-term survival prediction in breast cancer patients after radical surgery.

## Introduction

According to the latest 2022 global cancer statistics released by the International Agency for Research on Cancer (IARC), female breast cancer has a global incidence rate of 11.6%, making it the second most common cancer worldwide, following lung cancer (12.4%). Breast cancer is also the most frequently diagnosed cancer and the leading cause of cancer-related deaths among women globally (1). By 2040, the burden of breast cancer is projected to increase to over 3 million new cases and 1 million deaths annually (2). In China, approximately 357,200 new cases of female breast cancer were reported in 2022, ranking it as the second most common cancer among women (3). Despite significant advances in multimodal treatment strategies, including surgery, chemotherapy, and radiotherapy, prognosis remains highly variable among patients. This heterogeneity underscores the urgent need for reliable prognostic biomarkers to facilitate risk stratification and personalized postoperative management.

A growing body of evidence indicates a close association between metabolic disorders and cancer prognosis (4, 5). Specifically, insulin resistance (IR) has been implicated in the pathogenesis and progression of various cancers, including breast cancer (6, 7). IR leads to glucose intolerance and compensatory hyperinsulinemia, which can promote tumor cell proliferation, inhibit apoptosis, and enhance metastatic potential through pathways such as the insulin-like growth factor-1 (IGF-1) and the phosphatidylinositol 3 -kinase/akstrain transforming/mechanistic target of rapamycin (PI3K/AKT/mTOR) signaling cascade (8–11). Although the hyperinsulinemic-euglycemic clamp remains the gold standard for assessing IR, its clinical application is limited by complexity, cost, and invasiveness (12). Consequently, readily accessible surrogate indices derived from routine laboratory parameters, such as the atherogenic index of plasma (AIP) and the triglyceride-glucose (TyG) index, have been developed and validated (13, 14).

The AIP, calculated as log_10_(triglycerides [TG]/high density lipoprotein cholesterol [HDL-C]), is a well-established marker for assessing cardiovascular disease risk and IR (15, 16). Studies indicate that higher AIP values are associated with an increased risk of related cardiovascular events (17). AIP has also been linked to various metabolic disorders, including diabetes, metabolic syndrome, and non-alcoholic fatty liver disease (NAFLD) (13, 18, 19). Notably, Tagoe et al. reported significantly elevated AIP levels in breast cancer patients compared to healthy controls (20), suggesting a potential metabolic link in breast cancer pathogenesis.

Meanwhile, the TyG index, calculated as ln(TG× fasting plasma glucose [FPG]/2), has garnered increasing attention as a robust and accessible surrogate indicator of IR (21, 22). The TyG index and its related indicators have been closely associated with a variety of diseases, including metabolic disorders, NAFLD, cardiovascular diseases, and post-Corona Virus Disease (COVID)-19 syndrome (23–26). For instance, a large prospective study with a 12-year follow-up demonstrated that the TyG index was superior to the homeostasis model assessment of IR (HOMA-IR) in predicting the incidence of metabolic syndrome, underscoring its utility in identifying core metabolic dysfunctions (23). Moreover, a systematic review indicated that the TyG index serves as a significant predictor for the development of cardiovascular conditions such as coronary artery disease, heart failure, and arterial stiffness, highlighting its importance in cardiovascular risk stratification (25). More recently, the TyG index has emerged as a valuable marker in acute and post-acute health crises. It has been shown to be a strong independent risk factor for critical illness and mortality in patients with COVID-19, and is positively associated with the risk of post-COVID-19 syndrome, suggesting a link between IR and long-term sequelae of severe infection (26, 27).

While the TyG index’s association with a wide range of diseases is well-documented, its application in breast cancer has been relatively narrow. Most studies have focused on its utility for assessing disease risk (28, 29), with limited data on its prognostic value for long-term survival after diagnosis. Therefore, this study aims to retrospectively investigate the association between four IR surrogate indices (AIP, TyG, TyG-BMI, and METS-IR) and long-term overall survival (OS) and disease-free survival (DFS) in primary breast cancer patients who underwent radical surgery. Using a comprehensive analytical framework, we compared their independent prognostic value to identify the optimal indicator for risk stratification in the long-term management of postoperative breast cancer patients.

## Methods

### Study population

**Figure 1:**
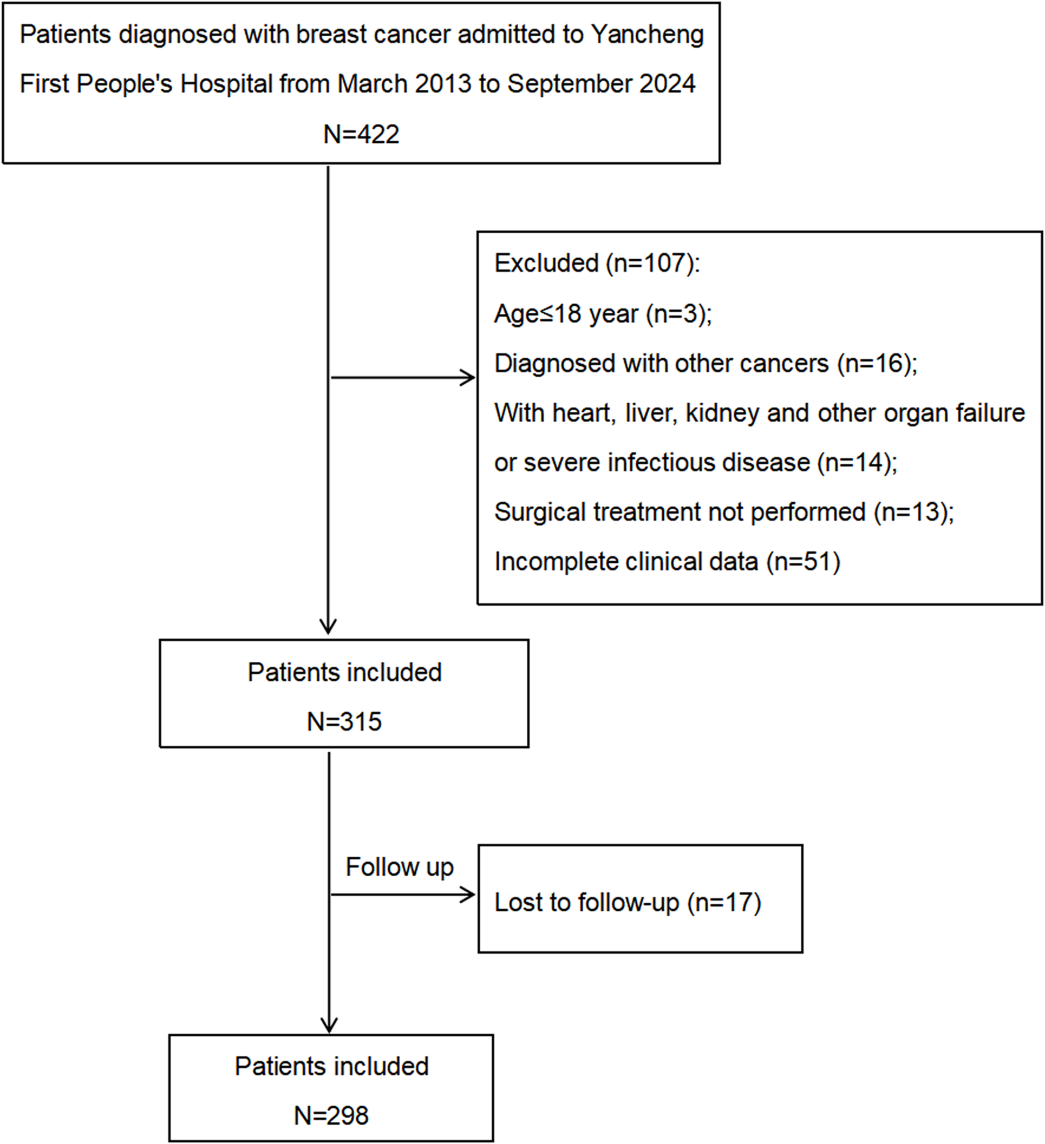
The flow diagram of sample selection in the study.

This retrospective study initially enrolled 422 female patients with primary breast cancer at Yancheng First People’s Hospital between March 2013 and September 2024. The following exclusion criteria were applied: (1) age under 18 years (n = 3); (2) history of other malignancies (n = 16); (3) presence of organ failure (e.g., cardiac, hepatic, or renal) or severe infectious diseases (n = 14); (4) breast cancer patients who did not receive surgical treatment (n = 13); (5) missing core laboratory parameters required for calculating IR indices such as AIP and TyG (including TG, HDL-C, and FPG) or key clinicopathological variables (such as histological grade, estrogen receptor (ER) status, progesterone receptor (PR) status et al) (n = 51); and (6) loss to follow-up preventing collection of survival outcome variables (n = 17). Ultimately, 298 female breast cancer patients who underwent surgery were included in the final analysis (**Figure 1**).

### Data collection

Patient information was collected from inpatient and outpatient electronic medical record systems. The data included demographic characteristics (age, height, weight, and marital status), past medical history (hypertension, diabetes, and hyperlipidemia), histological grade, ER status, PR status, human epidermal growth factor receptor 2 (HER2) status, Ki67 score, T stage, N stage, Karnofsky Performance Status (KPS) score, and laboratory parameters. Marital status was categorized as married or unmarried, with the latter including single, divorced, separated, and widowed patients. Baseline hematological parameters were obtained from standardized complete blood count (CBC) and biochemical tests performed within one week before surgery. These parameters included white blood cell count (WBC), red blood cell count (RBC), lymphocyte count, hemoglobin level, TG, total cholesterol (TC), HDL-C, low-density lipoprotein cholesterol (LDL-C), FPG and albumin level.

Body mass index (BMI) was calculated as weight in kilograms divided by the square of height in meters. The following formulas were used to calculate surrogate indices of insulin resistance (IR): AIP = log_10_(TG/HDL-C) (19), TyG = ln(TG × FPG/2), TyG-BMI = TyG × BMI, METS-IR = ln(2 × FPG + TG) × BMI/lnHDL-C (14).

### Outcomes

Follow-up data were obtained through the hospital outpatient system or via telephone interviews. Patients were evaluated every six months during the first two years after surgery, and annually thereafter. The cutoff date for follow-up was June 1, 2025. OS was defined as the time interval from the date of surgery to the date of death from any cause or the last follow-up. DFS was defined as the time interval from the date of surgery to the date of recurrence, metastasis, or death from any cause.

### Statistical analysis

Continuous variables with normal distribution are presented as mean ± standard deviation (SD), while skewed data are summarized as median and interquartile range (IQR). Categorical variables are expressed as counts and percentages (%). All analyses were performed using R software (version 4.4.2). A two-sided p-value < 0.05 was considered statistically significant.

The multivariable Cox proportional hazards models were employed to estimate hazard ratios (HRs) and 95% confidence intervals (CIs) for the association between the IR indices and both OS and DFS in breast cancer. Model 1 was unadjusted. Model 2 was adjusted for age, BMI, and marital status. Model 3 included all covariates in model 2, with additional adjustments for KPS score, past medical history (hypertension, diabetes, and hyperlipidemia), key tumor characteristics (histological grade, ER status, PR status, HER2 status, Ki67 score, T stage, and N stage), baseline hematological parameters (WBC, RBC, neutrophil percentage, lymphocyte percentage, monocyte percentage, hemoglobin, platelet count, TC and LDL-C). The IR indices were analyzed both as continuous variables (per 1-SD increase) and as categorical variables based on tertiles, with the lowest tertile (T1) serving as the reference group. The P-value for trend was calculated to assess the trend in risk associated with per 1-SD increase in the IR indices.

Kaplan-Meier survival analyses were used to visualize and compare survival probabilities over time among patients stratified by tertiles of each IR index. The log-rank tests were applied to evaluate the statistical significance of differences between survival curves. Restricted cubic spline (RCS) plots were employed to characterize potential nonlinear associations between the IR indices and both OS and DFS in breast cancer. The spline curves were constructed with four knots placed at the 5th, 35th, 65th, and 95th percentiles. A significant overall association (P < 0.05) with nonsignificant nonlinearity (P ≥ 0.05) indicated a linear relationship, whereas significant nonlinearity (P < 0.05) suggested a nonlinear association.

The area under the ROC curve (AUC) was calculated to evaluate the discriminative ability of each IR index for predicting 3-, 5-, and 10-year survival. An AUC of 0.5 indicates no discriminative ability, whereas 1.0 represents perfect discrimination. DeLong’s test was used to compare the AUC values among different indices. In these comparisons, the TyG index was set as the reference, and a P-value < 0.05 indicated a statistically significant difference in AUC values between the indices being compared.

Random Survival Forest (RSF), a machine learning method, was employed to complement traditional regression analyses. Using the variable importance measure derived from the RSF, we ranked the relative predictive power of metabolic-related variables, including the IR indices, for OS and DFS. This provided an alternative, data-driven evaluation of the factors most strongly associated with survival outcomes.

To assess the consistency of the associations between the primary IR indices (AIP and TyG) and survival, we conducted stratified Cox regression analyses across various patient subgroups. These subgroups were defined by age, BMI, marital status, history of chronic diseases, tumor stage, and other relevant clinical characteristics. Interaction terms between the subgrouping variables and the IR index were tested to determine whether the association varied significantly across subgroups.

## Results

### Baseline characteristics

The demographic and clinicopathological characteristics of the patients are summarized in **Table 1**. The mean age of the patients was 53.73 years, and the mean BMI was 24.57 kg/m². The prevalence rates of hypertension, diabetes, and hyperlipidemia were 17.45%, 10.07%, and 6.04%, respectively. Postoperative histological grading revealed that 123 patients (41.28%) were classified as stage II and 157 patients (52.68%) as stage III. Among these patients, the rates of ER, PR, and HER2 positivity were 62.75%, 50.67%, and 41.95%, respectively, while high Ki67 expression was observed in 70.47% of the cases.

**Table 1:** Baseline characteristics of the study population.

Variables	Total (n = 298)	Variables	Total (n = 298)
Age, year	53.73 ± 10.02	N stage, n(%)	
BMI, kg/m^2^	24.57 ± 3.39	N0	98 (32.89)
Marital status, n(%)		N1	103 (34.56)
Divorced	40 (13.42)	N2	46 (15.44)
Married	258 (86.58)	N3	51 (17.11)
KPS score	80.00 (80.00, 90.00)	Hemoglobin, g/L	117.40 ± 15.68
Hypertension, n(%)		Platelet, 10^9/L	195.78 ± 79.10
No	246 (82.55)	TG, mmol/L	1.96 ± 1.15
Yes	52 (17.45)	TC, mmol/L	5.06 ± 1.04
Diabetes, n(%)		HDL-C, mmol/L	1.21 ± 0.26
No	268 (89.93)	LDL-C, mmol/L	3.12 ± 0.75
Yes	30 (10.07)	FPG, mmol/L	5.60 ± 1.64
Hyperlipemia, n(%)		Total protein, g/L	67.77 ± 6.29
No	280 (93.96)	Albumin, g/L	40.09 ± 3.79
Yes	18 (6.04)	Prealbumin, g/L	227.34 ± 48.91
Histological grade, n(%)		WBC, 10^9/L	4.63 (3.70, 5.99)
I	18 (6.04)	Neutrophils, 10^9/L	2.59 (2.02, 3.64)
II	123 (41.28)	Neutrophil percentage, %	0.59 (0.51, 0.66)
III	157 (52.68)	Lymphocytes, 10^9/L	1.27 (0.95, 1.68)
ER status, n(%)		Lymphocyte percentage, %	0.30 (0.22, 0.36)
Negative	111 (37.25)	Monocytes, 10^9/L	0.39 (0.27, 0.49)
Positive	187 (62.75)	Monocytes percentage, %	0.08 (0.06, 0.10)
PR status, n(%)		RBC, 10^12/L	4.00 (3.60, 4.29)
Negative	147 (49.33)	Outcomes	
Positive	151 (50.67)	Survival, n(%)	240 (80.5)
HER2 status, n(%)		Death, n(%)	58 (19.5)
Negative	173 (58.05)	TyG	8.92 ± 0.59
Positive	125 (41.95)	TyG-BMI	219.03 ± 33.22
Ki67 score, n(%)		AIP	0.16 ± 0.27
<30	88 (29.53)	METS-IR	37.99 ± 6.24
≥30	210 (70.47)		
T stage, n(%)			
T1	114 (38.26)		
T2	144 (48.32)		
T3	33 (11.07)		
T4	7 (2.35)		

BMI, body mass index; KPS, karnofsky performance status; ER, estrogen receptor; PR, progesterone receptor; HER2, human epidermal growth factor receptor 2; TG, triglycerides; TC, total cholesterol; HDL-C, high-density lipoprotein cholesterol; LDL-C, low-density lipoprotein cholesterol; FPG, fasting plasma glucose; WBC, white blood cell count; RBC, red blood cell count; TyG, triglyceride-glucose index; TyG-BMI, TyG with body mass index; AIP, atherogenic index of plasma; METS-IR, metabolic score for insulin resistance.

The median follow-up for OS was 9.6 years, and the median follow-up for DFS was 8.2 years. By the end of the follow-up period (June 2025), 58 patients (19.5%) had died. The mean values of IR surrogate indexes (TyG, TyG-BMI, AIP, and METS-IR) were 8.92, 219.03, 0.16, and 37.99, respectively.

### Associations between IR surrogate indices and OS/DFS in breast cancer

We utilized multivariate Cox regression analysis to assess the associations between four IR indices and OS as well as DFS in breast cancer. As presented in **Table 2**, which summarizes the associations with OS, only the highest tertiles of the AIP and TyG index showed statistically significant differences in HRs and 95%CIs compared to the lowest tertiles (T1) across all three models (P < 0.05). In the fully adjusted model (model 3), the HRs (95% CIs) for T3 versus T1 of AIP and TyG were 2.28 (1.08-4.80) and 2.82 (1.26-6.33), respectively. Furthermore, per 1-SD increases in AIP and TyG, the risks of OS were elevated in all three models. Specifically, in model 3, per 1-SD increases in AIP and TyG were associated with a 50% and 55% increase in risk, respectively, and these trends were statistically significant (P for trend < 0.05). Although TyG-BMI and METS-IR also exhibited statistically significant trends toward increased risk per 1-SD increases in models 2 and 3 (P for trend < 0.05), comparisons of HRs across tertiles did not reach statistical significance, suggesting weaker associations between TyG-BMI or METS-IR and OS.

**Table 2:** Multivariate Cox regression analysis of the associations between IR surrogate indexes and OS in breast cancer patients.

Variables	No. of deaths	Model 1		Model 2		Model 3	
		HR (95%CI)	*P* value	HR (95%CI)	*P* value	HR (95%CI)	*P* value
AIP Tertiles							
T1	16	1.00 (Reference)		1.00 (Reference)		1.00 (Reference)	
T2	18	1.56 (0.79 ~ 3.07)	0.201	1.52 (0.77 ~ 3.00)	0.233	1.40 (0.63 ~ 3.13)	0.415
T3	24	2.19 (1.16 ~ 4.15)	**0.016**	2.18 (1.15 ~ 4.14)	**0.017**	2.28 (1.08 ~ 4.80)	**0.030**
Per 1-SD increase		1.44 (1.10 ~ 1.87)		1.43 (1.10 ~ 1.87)		1.50 (1.11 ~ 2.02)	
*P* for trend			**0.007**		**0.008**		**0.008**
TyG Tertiles							
T1	12	1.00 (Reference)		1.00 (Reference)		1.00 (Reference)	
T2	23	2.36 (1.17 ~ 4.75)	**0.016**	2.32 (1.15 ~ 4.68)	**0.019**	2.13 (0.93 ~ 4.85)	0.072
T3	23	2.63 (1.30 ~ 5.31)	**0.007**	2.56 (1.26 ~ 5.22)	**0.009**	2.82 (1.26 ~ 6.33)	**0.012**
Per 1-SD increase		1.46 (1.13 ~ 1.89)		1.46 (1.13 ~ 1.89)		1.55 (1.15 ~ 2.10)	
*P* for trend			**0.004**		**0.004**		**0.004**
TyG-BMI Tertiles							
T1	21	1.00 (Reference)		1.00 (Reference)		1.00 (Reference)	
T2	18	0.95 (0.51 ~ 1.79)	0.876	1.27 (0.58 ~ 2.78)	0.543	0.86 (0.36 ~ 2.09)	0.743
T3	19	1.06 (0.57 ~ 1.97)	0.864	1.92 (0.66 ~ 5.63)	0.234	1.53 (0.45 ~ 5.24)	0.496
Per 1-SD increase		1.08 (0.83 ~ 1.40)		2.28 (1.26 ~ 4.11)		2.61 (1.31 ~ 5.19)	
*P* for trend			0.551		**0.006**		**0.006**
METS-IR Tertiles							
T1	23	1.00 (Reference)		1.00 (Reference)		1.00 (Reference)	
T2	16	0.86 (0.45 ~ 1.64)	0.646	1.03 (0.49 ~ 2.20)	0.931	0.93 (0.40 ~ 2.16)	0.863
T3	19	0.97 (0.53 ~ 1.79)	0.933	1.42 (0.54 ~ 3.78)	0.479	1.62 (0.53 ~ 4.97)	0.400
Per 1-SD increase		1.16 (0.88 ~ 1.52)		2.12 (1.32 ~ 3.40)		2.09 (1.24 ~ 3.52)	
*P* for trend			0.294		**0.002**		**0.006**

Bold values indicate *P* < 0.05. Model 1: crude; Model 2: adjusted for age, BMI, marital status; Model 3: adjusted for age, BMI, marital status, KPS score, hypertension, diabetes, hyperlipemia, histological grade, ER status, PR status, HER2 status, Ki67 score, T stage, N stage, WBC, neutrophil percentage, lymphocyte percentage, monocytes percentage, RBC, hemoglobin, platelet, TC, LDL-C.

OS, overall survival; IR, insulin resistance; HR, hazard ratio; CI, confidence interval; AIP, atherogenic index of plasma; TyG, triglyceride-glucose index; TyG-BMI, TyG with body mass index; METS-IR, metabolic score for insulin resistance; SD, standard deviation.

**Table 3** presents the associations between the IR indices and DFS. For DFS, the TyG index emerged as the most consistent predictor, indicating statistically significant associations across all three analytical measures (tertile comparisons, per 1-SD increases, and trend tests). AIP also showed significant trends and elevated HRs per 1-SD increase in all three models; however, in the model 3, the comparison between the highest (T3) and lowest tertiles (T1) was of borderline significance (P = 0.055), suggesting a more gradual increase in risk. Consistent with the OS findings, TyG-BMI and METS-IR exhibited significant trends in models 2 and 3 for DFS, yet no significant associations were observed in tertile-based comparisons, again indicating less distinct risk stratification.

**Table 3:** Multivariate Cox regression analysis of the associations between IR surrogate indexes and DFS in breast cancer patients.

Variables	No. of deaths	Model 1		Model 2		Model 3	
		HR (95%CI)	*P* value	HR (95%CI)	*P* value	HR (95%CI)	*P* value
AIP Tertiles							
T1	16	1.00 (Reference)		1.00 (Reference)		1.00 (Reference)	
T2	18	1.39 (0.71 ~ 2.74)	0.336	1.34 (0.68 ~ 2.65)	0.398	1.27 (0.59 ~ 2.75)	0.547
T3	24	2.00 (1.05 ~ 3.79)	**0.034**	2.04 (1.07 ~ 3.87)	**0.030**	2.02 (0.99 ~ 4.14)	0.055
Per 1-SD increase		1.41 (1.08 ~ 1.85)		1.42 (1.09 ~ 1.86)		1.49 (1.11 ~ 2.01)	
*P* for trend			**0.012**		**0.011**		**0.008**
TyG Tertiles							
T1	12	1.00 (Reference)		1.00 (Reference)		1.00 (Reference)	
T2	23	2.15 (1.07 ~ 4.33)	**0.033**	2.16 (1.07 ~ 4.37)	**0.032**	1.99 (0.90 ~ 4.37)	0.087
T3	23	2.34 (1.16 ~ 4.71)	**0.018**	2.41 (1.19 ~ 4.90)	**0.015**	2.49 (1.11 ~ 5.60)	**0.028**
Per 1-SD increase		1.49 (1.14 ~ 1.94)		1.50 (1.15 ~ 1.96)		1.58 (1.15 ~ 2.15)	
*P* for trend			**0.003**		**0.003**		**0.004**
TyG-BMI Tertiles							
T1	21	1.00 (Reference)		1.00 (Reference)		1.00 (Reference)	
T2	18	0.86 (0.46 ~ 1.62)	0.650	1.19 (0.54 ~ 2.64)	0.662	0.83 (0.35 ~ 1.95)	0.661
T3	19	1.04 (0.56 ~ 1.93)	0.907	1.96 (0.66 ~ 5.79)	0.225	1.56 (0.46 ~ 5.23)	0.473
Per 1-SD increase		1.08 (0.83 ~ 1.40)		2.45 (1.33 ~ 4.50)		2.76 (1.36 ~ 5.61)	
*P* for trend			0.586		**0.004**		**0.005**
METS-IR Tertiles							
T1	23	1.00 (Reference)		1.00 (Reference)		1.00 (Reference)	
T2	16	0.76 (0.40 ~ 1.45)	0.408	0.89 (0.42 ~ 1.91)	0.770	0.86 (0.37 ~ 1.97)	0.716
T3	19	0.93 (0.51 ~ 1.71)	0.814	1.27 (0.48 ~ 3.39)	0.632	1.45 (0.48 ~ 4.39)	0.505
Per 1-SD increase		1.10 (0.84 ~ 1.44)		1.94 (1.19 ~ 3.16)		2.05 (1.21 ~ 3.49)	
*P* for trend			0.482		**0.008**		**0.008**

Bold values indicate *P* < 0.05. Model 1: crude; Model 2: adjusted for age, BMI, marital status; Model 3: adjusted for age, BMI, marital status, KPS score, hypertension, diabetes, hyperlipemia, histological grade, ER status, PR status, HER2 status, Ki67 score, T stage, N stage, WBC, neutrophil percentage, lymphocyte percentage, monocytes percentage, RBC, hemoglobin, platelet, TC, LDL-C.

DFS, disease-free survival; IR, insulin resistance; HR, hazard ratio; CI, confidence interval; AIP, atherogenic index of plasma; TyG, triglyceride-glucose index; TyG-BMI, TyG with body mass index; METS-IR, metabolic score for insulin resistance; SD, standard deviation.

**Figure 2:**
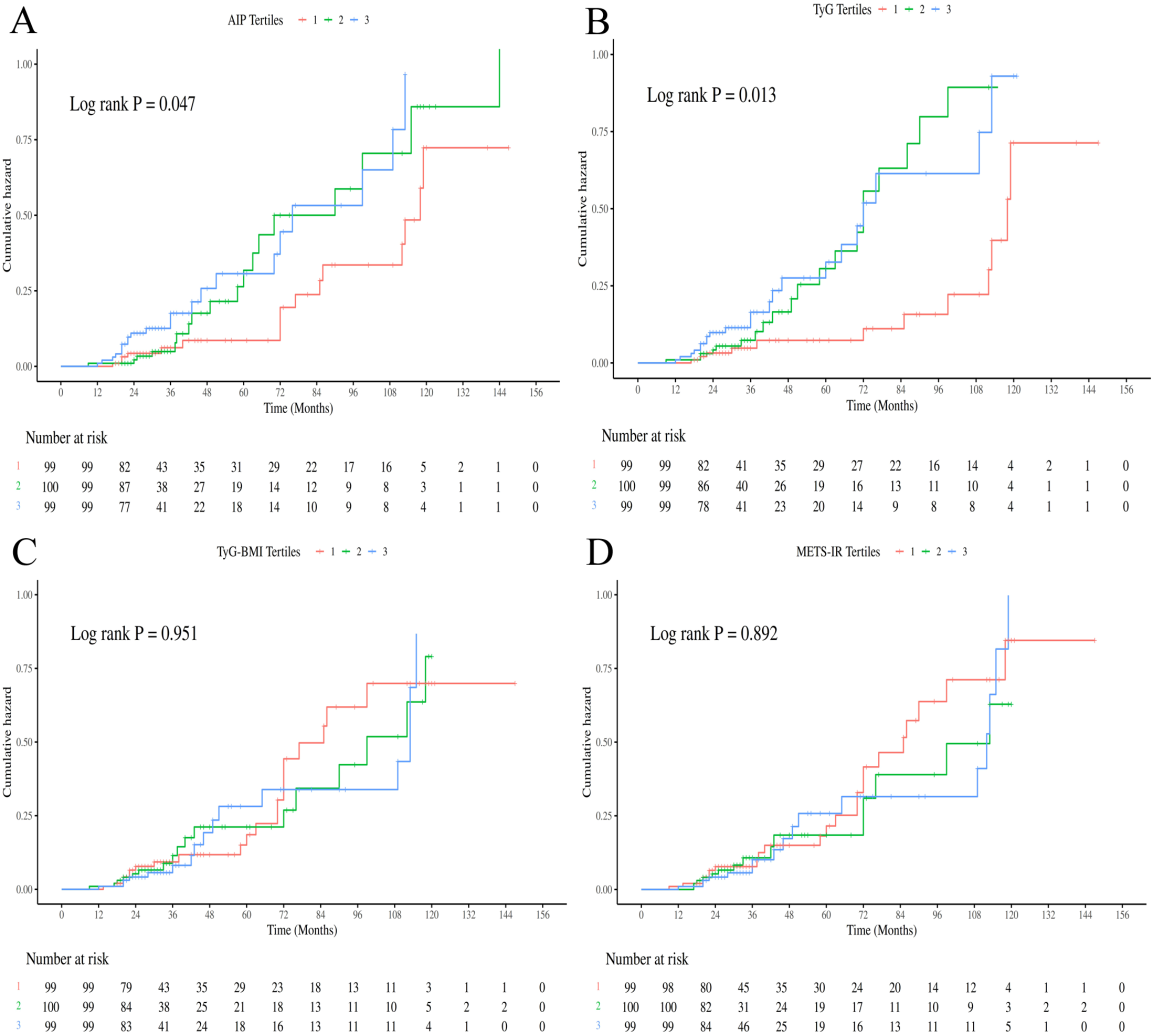
Kaplan-Meier curves of OS divided by tertiles of IR surrogate indexes [**(A)** AIP; **(B)** TyG; **(C)** TyG-BMI; **(D)** METS-IR].

**Figure 3:**
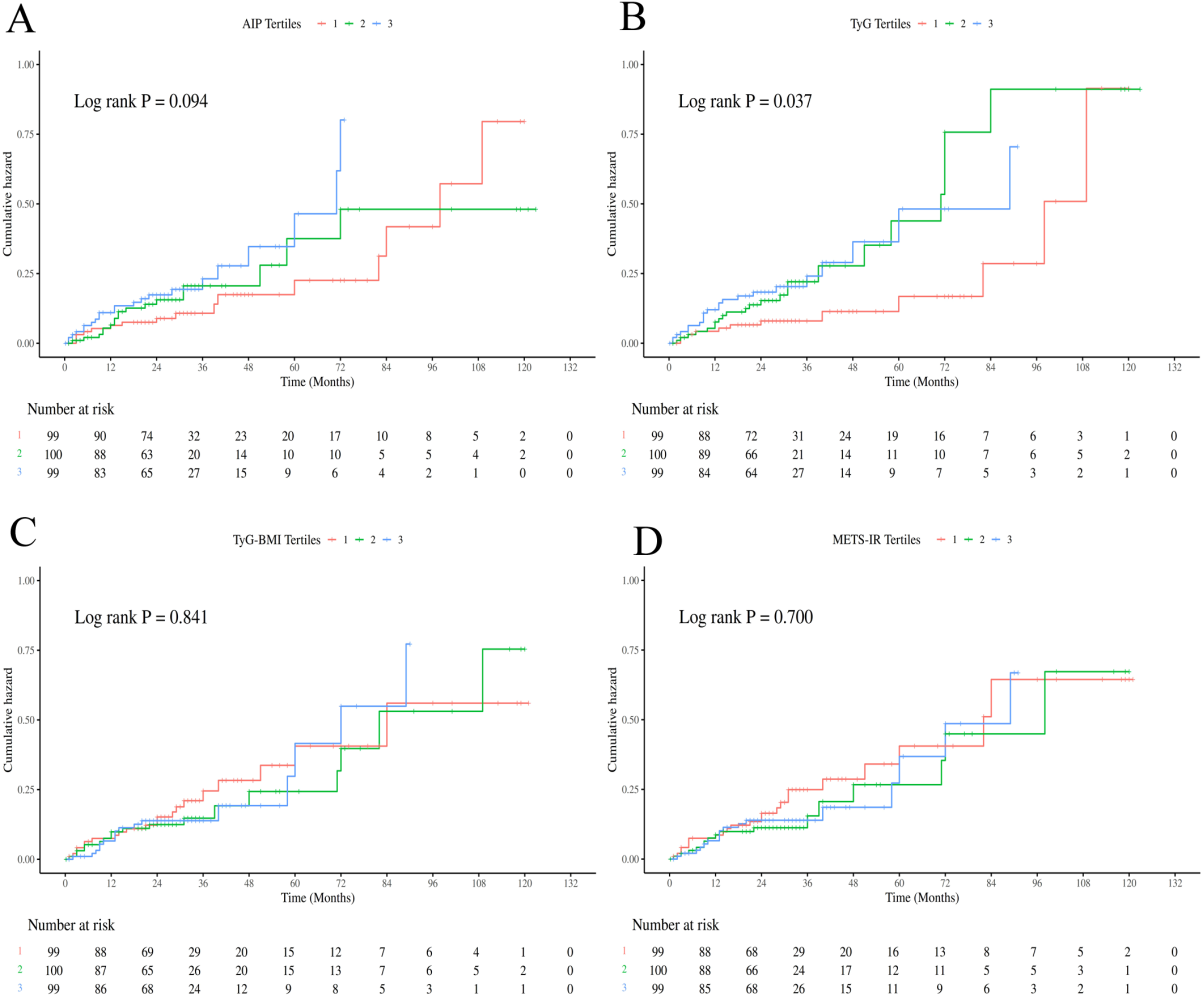
Kaplan-Meier curves of DFS divided by tertiles of IR surrogate indexes [**(A)** AIP; **(B)** TyG; **(C)** TyG-BMI; **(D)** METS-IR].

**Figure 4:**
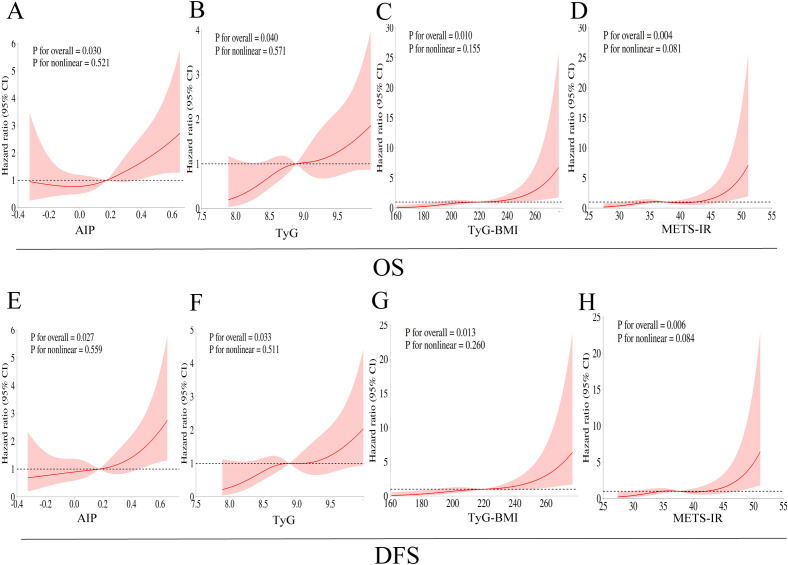
Restricted cubic spline curves for analyzing the nonlinear relationships between IR surrogate indexes and OS and DFS of breast cancer patients [**(A, E)** AIP; **(B, F)** TyG; **(C, G)** TyG-BMI; **(D, H)** METS-IR]. Hazard ratios were adjusted for age, BMI, marital status, KPS score, hypertension, diabetes, hyperlipemia, histological grade, ER status, PR status, HER2 status, Ki67 score, T stage, N stage, WBC, neutrophil percentage, lymphocyte percentage, monocytes percentage, RBC, hemoglobin, platelet, TC, LDL-C.

Consistent with the Cox regression analyses, Kaplan-Meier survival curves showed that patients in the highest tertile of TyG had significantly worse OS and DFS compared to those in the lowest tertile (log-rank test, P < 0.05; **Figure 2B**, **Figure 3B**). For AIP, a statistically significant difference was observed only in OS (**Figure 2A**), not in DFS (**Figure 3A**). In contrast, TyG-BMI and METS-IR showed no significant association with either OS or DFS (**Figures 2C, D**, **Figures 3C, D**). RCS analyses revealed linear relationships between all four IR indices and both OS and DFS after adjusting for covariates (P for nonlinearity > 0.05; **Figures 4A-H**).

### Predictive value of IR surrogate indices for breast cancer survival

**Figure 5:**
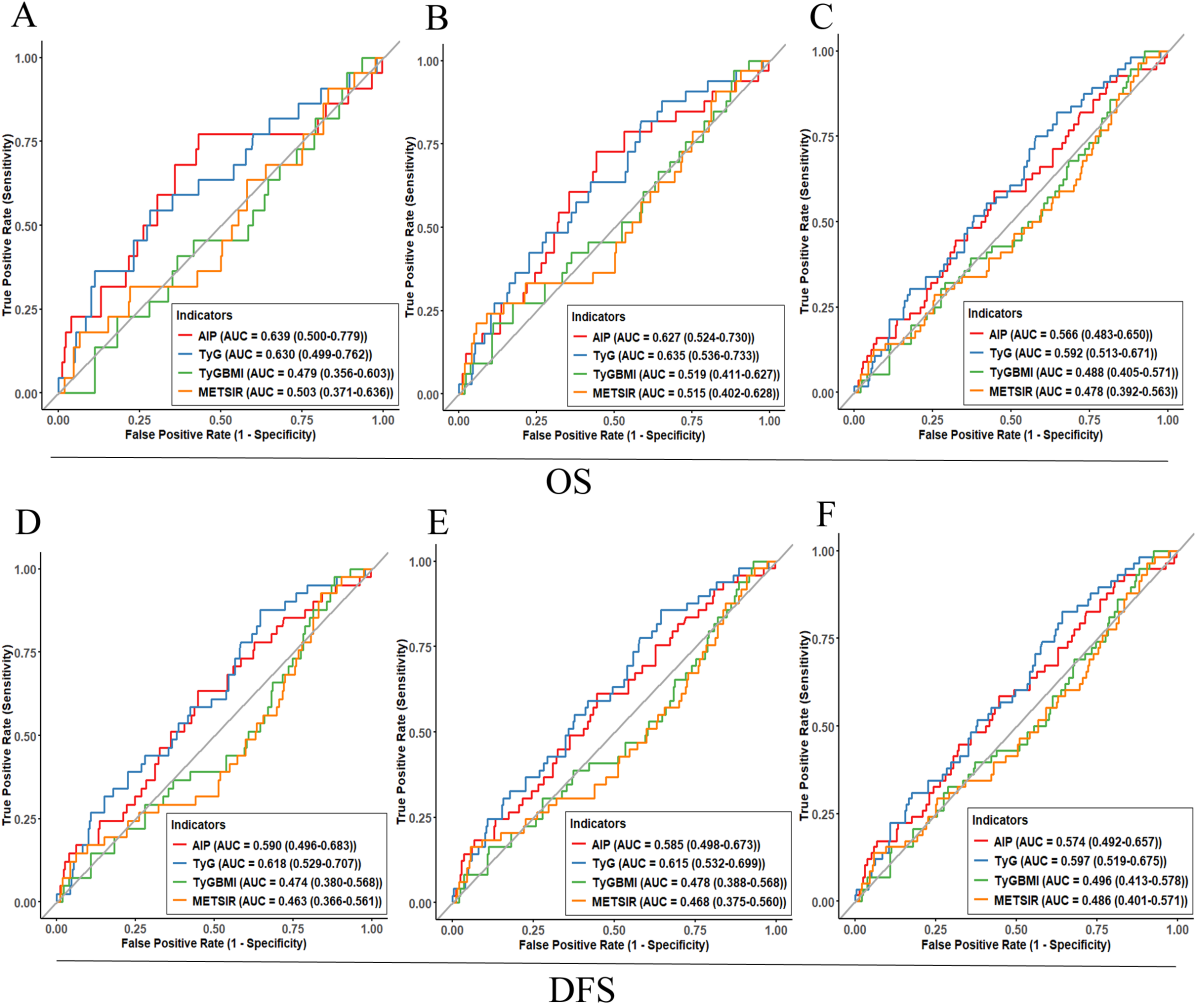
The receiver operating characteristic (ROC) curve analysis of IR surrogate indexes for predicting OS and DFS of breast cancer [**(A)** 3-year OS; **(B)** 5-year OS; **(C)** 10-year OS; **(D)** 3-year DFS; **(E)** 5-year DFS; **(F)** 10-year DFS].

**Figure 5** and **Table 4** present the predictive performance of the IR surrogate indices for 3-, 5-, and 10-year OS and DFS in breast cancer. AIP and TyG showed superior predictive ability for 3-, 5-, and 10-year OS and DFS compared to TyG-BMI and METS-IR (**Figures 5A-F**). Statistical comparison of the AUCs indicated that the predictive performance of AIP and TyG was not significantly different from each other at any time point (P > 0.05). However, both AIP and TyG generally showed higher AUC values compared to TyG-BMI and METS-IR (P < 0.05, **Table 4**). It is important to note that the discriminative ability of AIP and TyG was modest, with the lower bounds of the 95% CIs for AUCs approaching 0.5.

**Table 4:** Comparisons of the AUCs of IR surrogate indexes in predicting OS and DFS.

Variables	AUC (95%CI)	*P* value	Variables	AUC (95%CI)	*P* value
OS			DFS		
3-year			3-year		
TyG	0.630(0.499-0.762)	1.00 (Reference)	TyG	0.618(0.529-0.707)	1.00 (Reference)
AIP	0.639(0.500-0.779)	0.823	AIP	0.590(0.496-0.683)	0.324
TyG-BMI	0.479(0.356-0.603)	**0.018**	TyG-BMI	0.474(0.380-0.568)	**0.007**
METS-IR	0.503(0.371-0.636)	0.053	METS-IR	0.463(0.366-0.561)	**0.004**
5-year			5-year		
TyG	0.635(0.536-0.733)	1.00 (Reference)	TyG	0.615(0.532-0.699)	1.00 (Reference)
AIP	0.627(0.524-0.730)	0.813	AIP	0.585(0.498-0.673)	0.264
TyG-BMI	0.519(0.411-0.627)	**0.043**	TyG-BMI	0.478(0.388-0.568)	**0.004**
METS-IR	0.515(0.402-0.628)	**0.046**	METS-IR	0.468(0.375-0.560)	**0.002**
10-year			10-year		
TyG	0.592(0.513-0.671)	1.00 (Reference)	TyG	0.597(0.519-0.675)	1.00 (Reference)
AIP	0.566(0.483-0.650)	0.305	AIP	0.574(0.492-0.657)	0.351
TyG-BMI	0.488(0.405-0.571)	**0.023**	TyG-BMI	0.496(0.413-0.578)	**0.024**
METS-IR	0.478(0.392-0.563)	**0.015**	METS-IR	0.486(0.401-0.571)	**0.016**

Bold values indicate *P* < 0.05.

AUC, area under the receiver operating characteristic curve; IR, insulin resistance; OS, overall survival; DFS, disease-free survival; CI, confidence interval; TyG, triglyceride-glucose index; AIP, atherogenic index of plasma; TyG-BMI, TyG with body mass index; METS-IR, metabolic score for insulin resistance.

### Variable importance and subgroup analysis

**Figure 6:**
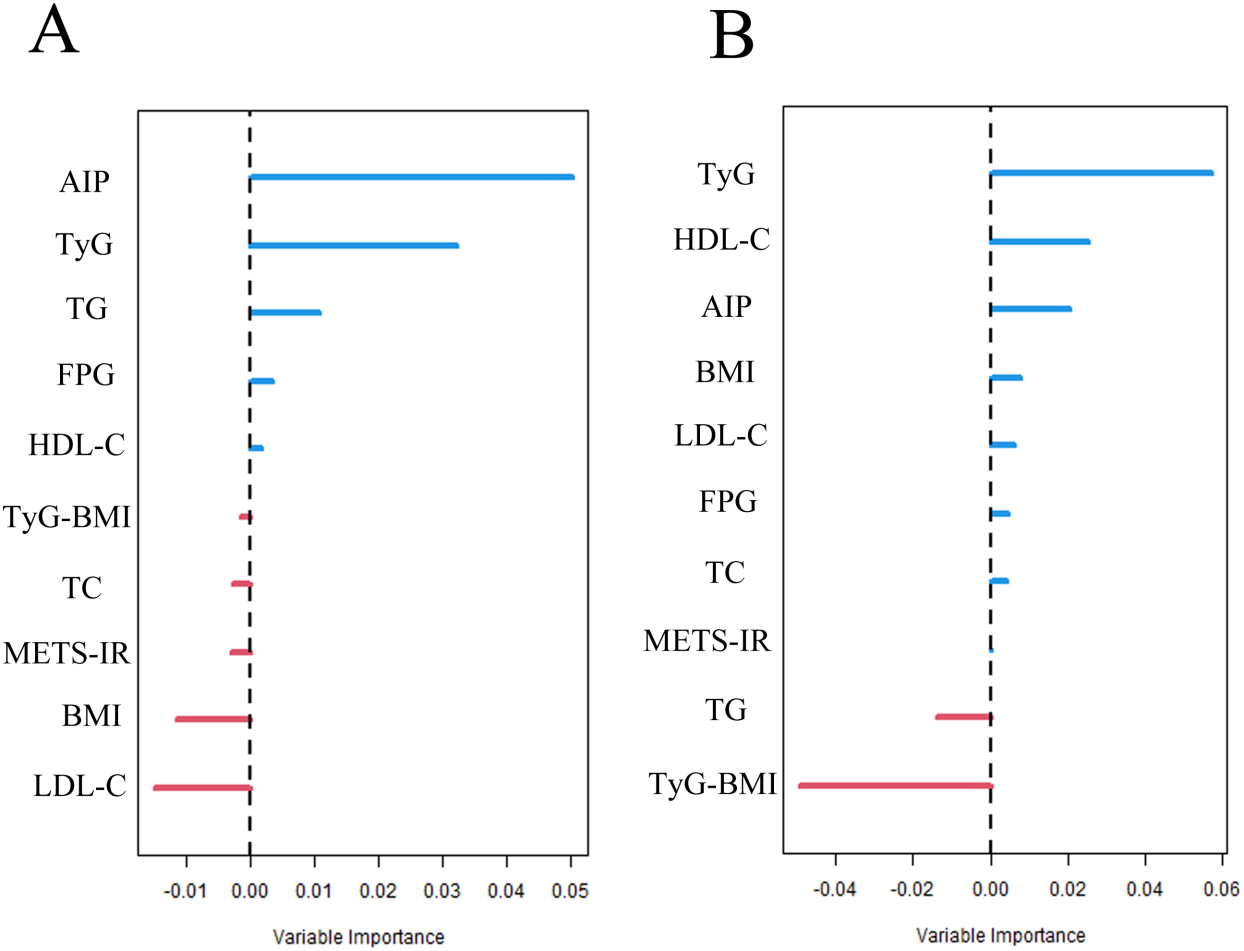
Random Survival Forests (RSF) for ranking variable importance [**(A)** OS; **(B)** DFS].

We ranked the importance of the IR surrogate indices and related variables using RSF analysis. As shown in **Figure 6A**, AIP was the most significant variable for OS in breast cancer, followed by TyG. For DFS (**Figure 6B**), TyG was the most important predictor, while AIP ranked third, following HDL-C.

**Figure 7:**
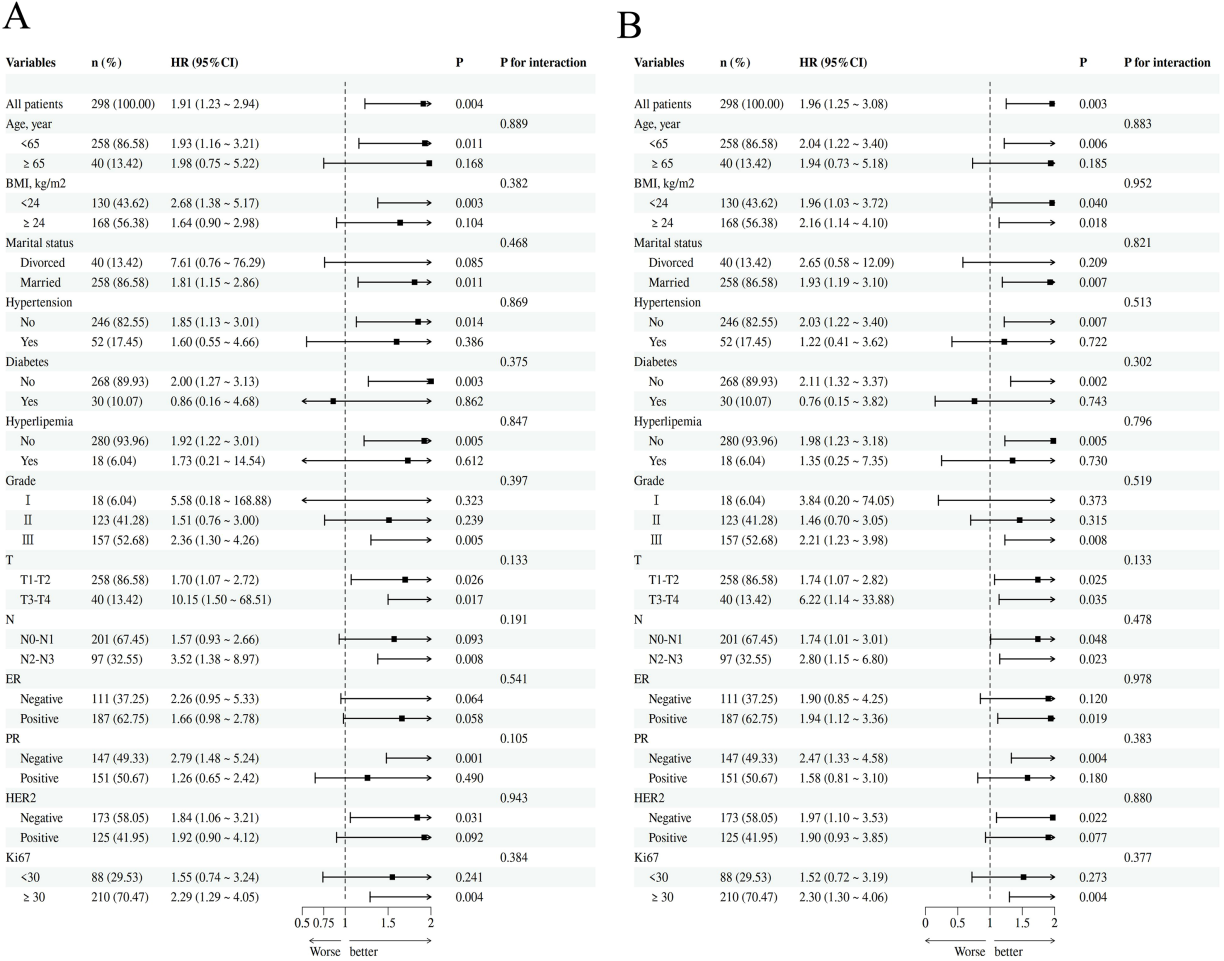
Subgroup analysis of the association between TyG and breast cancer OS and DFS [**(A)** OS; **(B)** DFS].

Subgroup analyses were performed to evaluate the associations of TyG and AIP with survival across different patient subgroups. Stratified by age, BMI, marital status, hypertension, diabetes, hyperlipidemia, and histological grade, et al, the results indicated that the associations between TyG and both OS (**Figure 7A**) and DFS (**Figure 7B**) remained consistent across all subgroups. Moreover, no significant interaction was observed between any subgroup variable and TyG in relation to breast cancer survival (P for interaction > 0.05). The subgroup analysis for AIP (**Supplementary Figure S1**) yielded results generally consistent with those observed for TyG.

## Discussion

This retrospective cohort study identifies the preoperative AIP and TyG indices as readily accessible and promising prognostic factors for long-term survival in postoperative breast cancer patients. The key finding is that elevated levels of these indices, which reflect underlying IR and atherogenic dyslipidemia, are associated with a higher risk of reduced OS. Notably, the TyG index also significantly predicted worse DFS. In contrast, the more complex indices that incorporated BMI (TyG-BMI and METS-IR) did not demonstrate superior prognostic value in our cohort.

The robust association of AIP and TyG with poor survival outcomes is strongly supported by a well-established pathophysiological framework linking metabolic dysregulation to cancer progression (30). Insulin resistance (IR), characterized by diminished cellular response to insulin, leads to compensatory hyperinsulinemia. This state of high insulin exerts direct and indirect carcinogenic effects (8). Specifically, hyperinsulinemia directly activates insulin receptors and the IGF-1/2 receptors on tumor cells, thereby stimulating the PI3K/AKT/mTOR and rat sarcoma/mitogen-activated protein kinase (RAS/MAPK) signaling pathways to further promote tumor cell growth and survival (11, 31).

Beyond hyperinsulinemia, the specific metabolic disturbances captured by AIP and TyG play a critical role. The TyG index, reflecting impaired glucose metabolism and elevated triglycerides, signifies a state of lipid oversupply. Cancer cells can exploit these circulating lipids for energy production via β-oxidation and as building blocks for membrane biosynthesis, supporting rapid tumor growth and proliferation (32). The AIP, calculated as the log of the TG-to-HDL-C ratio, more specifically captures an atherogenic lipid profile. A high AIP indicates an abundance of triglyceride-rich lipoproteins and small, dense LDL particles, alongside low levels of protective HDL. This dyslipidemic milieu is increasingly recognized as a key modifier of the tumor microenvironment (33). For instance, HDL dysfunction can impair reverse cholesterol transport from tumor cells, while oxidized LDL can infiltrate the tumor stroma and promote chronic inflammation and oxidative stress (32, 33). This inflammatory state, characterized by elevated levels of cytokines such as interleukin-6 (IL-6) and tumor necrosis factor-α (TNF-α), which in turn exacerbate oxidative stress and insulin resistance, thereby promoting tumorigenesis (34).

The consistent association of AIP and TyG with survival outcomes in Cox regression and Kaplan-Meier analyses, along with their high ranking in the RSF analysis, are notable findings. Although ROC analysis revealed that their AUCs indicated only moderate discriminative ability, they consistently outperformed the more complex indices TyG-BMI and METS-IR. This may be because TyG-BMI and METS-IR, despite incorporating BMI, might dilute the specific metabolic dysfunction signals driven by IR in the context of cancer survival. As supported by a meta-analysis indicating a U-shaped relationship between BMI and breast cancer survival (35), BMI alone may not adequately capture the specific metabolic risk relevant to cancer outcomes. In contrast, AIP and TyG are derived directly from core metabolic parameters (TG, HDL-C, and FPG) which more directly reflect the pathogenic lipid-glucose metabolic interplay and IR severity.

Tagoe et al. previously reported elevated AIP levels in breast cancer patients compared to healthy controls (20), suggesting an underlying metabolic link. A literature search revealed that AIP has rarely been studied in oncology, with only three publications reporting its ability to predict the risk of colorectal and prostate tumors (36–38). Our study extends existing literature by demonstrating that preoperative AIP levels are robustly associated with long-term survival after surgery, a finding scarcely reported in oncology. Similarly, while prior research has primarily utilized the TyG index to assess breast cancer risk (39–41), investigations into its prognostic value remain limited. A study by Li et al. involving 335 early breast cancer patients receiving neoadjuvant chemotherapy (NACT) found that a high pre-NACT TyG index was associated with worse DFS (HR = 2.402, P = 0.008) and OS (HR = 3.206, P = 0.010) (42). Our work further establishes the predictive utility of the TyG index for 3-, 5-, and 10-year OS and DFS in a surgical cohort, thereby extending the current literature and underscoring the impact of IR on cancer outcomes.

However, several limitations should be acknowledged. First, the retrospective, single-center design with a limited sample size, along with the direct deletion of missing data, inherently carries risks of selection bias and unmeasured confounding, despite our efforts to adjust for known confounding factors. Second, IR indices were measured only once preoperatively, preventing assessment of temporal fluctuations or postoperative metabolic changes. Third, detailed treatment information was not fully available, potentially leading to residual confounding. Fourth, the generalizability of our findings requires external validation in diverse populations and settings. Finally, the discriminative ability of the preoperative AIP and TyG indices, as measured by the AUC, was modest for predicting survival at specific time points. Future large-scale, multicenter prospective studies should incorporate dynamic monitoring of IR indices alongside other clinical indicators. These studies are needed to validate the current findings and to determine whether interventions targeting IR can improve survival outcomes.

## Conclusions

In conclusion, this study indicates that the preoperative AIP and TyG index are promising, cost-effective tools for risk stratification in breast cancer patients undergoing radical surgery. Their prognostic value, rooted in the fundamental biology of IR and lipid metabolism, appears consistent across patient subgroups. These findings highlight the critical role of metabolic health in breast cancer progression and underscore the potential of metabolic phenotyping in oncology. Future large-scale, multicenter prospective studies should incorporate dynamic monitoring of IR indices alongside other clinical indicators. These studies are needed to validate the current findings and to determine whether interventions targeting IR can improve survival outcomes.

## Data Availability

The raw data supporting the conclusions of this article will be made available by the authors, without undue reservation.

## Supplementary material

Supplementary Figure 1 Subgroup analysis of the association between AIP and breast cancer OS and DFS (**(A)** OS; **(B)** DFS).
