# Supplementary figures and images for: The atherogenic index of plasma and triglyceride-glucose index as promising predictors of overall and disease-free survival in postoperative breast cancer patients
Source: Front Endocrinol (Lausanne). 2026 Jan 6;16:1728451. doi: 10.3389/fendo.2025.1728451 (PMC12815778; doi:10.3389/fendo.2025.1728451)

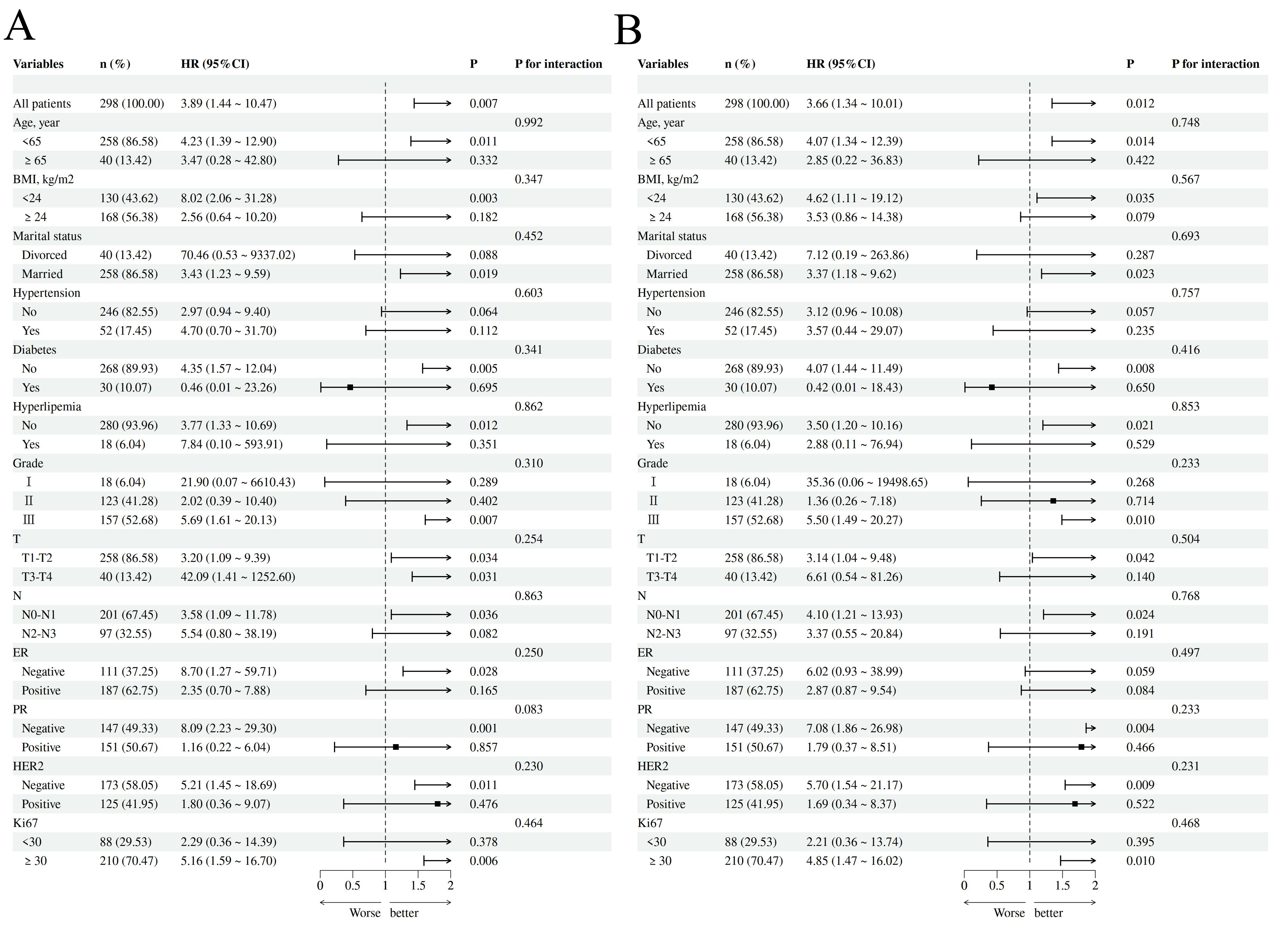

Supplement: Supplementary Figure 1 — Subgroup analysis of the association between AIP and breast cancer OS and DFS ((A) OS; (B) DFS). [file Image1.tif]
